# Comparison and Analysis of the Drug-Resistance Mechanism of Osimertinib- and Almonertinib-Resistant Cell Lines

**DOI:** 10.1155/ancp/5578693

**Published:** 2025-03-10

**Authors:** Chuangjie Zheng, Yingfang Ren, Ke Wang, Xinrong Chen, Jiahao Tao, Cuifen Zhang, Zeyu Liu, Lingling Sun, Linzhu Zhai

**Affiliations:** ^1^Cancer Center, The First Affiliated Hospital of Guangzhou University of Chinese Medicine 510405, Guangzhou, Guangdong, China; ^2^Lingnan Medical Research Center, Guangzhou University of Chinese Medicine, Guangzhou, Guangdong, China

**Keywords:** almonertinib, drug resistance, EGFR, EGFR-TKIs, osimertinib

## Abstract

**Background:** Non–small-cell lung cancer remains the leading cause of cancer-related deaths globally, and epidermal growth factor receptor mutations have been identified as crucial drivers of the disease. Encouragingly, epidermal growth factor receptor tyrosine kinase inhibitors have demonstrated promising clinical outcomes. Nonetheless, the emergence of resistance to third-generation EGFR-TKIs like osimertinib and almonertinib is an inevitable challenge.

**Methods:** In this study, we generated almonertinib-resistant cell lines from H-1975 and HCC827 lung cancer cell lines. We utilized various assays, including cell proliferation assays, hematoxylin and eosin staining, and cell cycle assays, to investigate the characteristics of drug-resistant cells. Additionally, we performed RNA transcriptome sequencing to identify differentially expressed genes (DEGs) in almonertinib-resistant cells. To further expand our analysis, we obtained sequencing data of osimertinib-resistant cells from the Gene Expression Omnibus (GEO) dataset and identified DEGs in these cells. We performed Gene Ontology (GO) and Kyoto Encyclopedia of Genes and Genomes (KEGG) analyses to assess the biological functions and signaling mechanisms associated with DEGs. Furthermore, the survival prognosis and immune cell infiltration of common differentially expressed genes (co-DEGs) in osimertinib—and almonertinib-resistant cells were analyzed, and the expression of a co-DEG (*IGFBP7*) was verified through quantitative reverse transcriptase polymerase chain reaction (qPCR) and western blotting (WB) assays. Gene knockdown plasmids were constructed for cell transfection, and the invasive ability of resistant cells was assessed using a Transwell assay following the knockdown of *IGFBP7*.

**Results:** Experimental cell counting kit–8 cytotoxicity studies revealed intriguing findings regarding drug resistance in lung cancer cells. Specifically, the IC_50_ values and resistance factors of H-1975 and HCC827 cells were found to be 1.9 nM and 833.58 and 2.2 nM and 631.95, respectively. In addition to these quantitative results, comparative observations of the cell morphology and cell cycle revealed significant alterations in drug-resistant cells. Transcriptome sequencing analysis identified 220 DEGs between H-1975 and H-1975/AR and 736 DEGs between HCC827 and HCC827/AR. Interestingly, screening of overlapping DEGs with osimertinib-resistant cells in the GEO database identified some common genes, such as *IGFBP7* and *RFTN1*, which were found to be associated with the improved prognosis of non–small–cell lung cancer by survival analysis. Furthermore, GO analysis and KEGG pathway enrichment analysis revealed different pathway changes in different drug-resistant cells. Survival analysis indicated that a higher expression of co-DEGs (*IGFBP7*, *RFTN1*) was associated with a more favorable prognosis. Furthermore, *IGFBP7* expression is strongly associated with infiltration levels of CD8+ T cells, Tregs, and macrophage cells in lung adenocarcinoma. Molecular biology experiments confirmed that the mRNA and protein expression level of *IGFBP7* were over-expressed in almonertinib-resistance cells. H-1975/AR cells were transfected with si-*IGFBP7*, and the results of transfection were verified at the mRNA and protein levels. After knocking down gene expression, the IC_50_ of the cells was 0.3 ± 0.02 µM, which was significantly lower than that of untransfected cells. Additionally, the invasion of cells in the knockdown group was repressed.

**Conclusions:** These findings indicated that almonertinib and osimertinib exhibited distinct resistance mechanisms in vitro, underscoring the need for tailored treatment approaches.

## 1. Introduction

Epidermal growth factor receptor (EGFR) is reported as an important driver oncogene in non–small-cell lung cancer (NSCLC) and patients with EGFR mutations, including exon 19 deletion and exon 21 L858R point mutation, initially respond well to the first- or second-generation of EGFR tyrosine kinase inhibitors (EGFR-TKIs) [1]. However, most patients could eventually develop acquired resistance after 9–12 months of treatment [2].

The most common mechanism of resistance in patients using either first- or second-generation EGFR-TKIs is a second-site T790M mutation of EGFR exon 20 [3]. The third-generation EGFR-TKIs, including osimertinib and almonertinib, were developed to overcome T790M mutation-mediated resistance [4, 5]. Both osimertinib and almonertinib led to superior progression-free survival (PFS) compared to the first-generation EGFR-TKIs (gefitinib or erlotinib) as first-line treatment in NSCLC patients with EGFR mutations [6, 7]. In particular, osimertinib led to a promising median PFS of 19 months, while almonertinib led to a median PFS of 19.3 months [8, 9]. However, with their widespread use in clinical practice, acquired resistance inevitably developed. Similar to first-generation EGFR-TKIs, the known resistance mechanisms of third-generation EGFR-TKIs primarily include EGFR secondary mutation, bypass activation, and histological transformation [10]. Among types of secondary mutant resistance to EGFR, the most common resistance mechanism is the C797X mutation, which occurs in up to 29% of resistant patients on osimertinib (after first-/second-line therapy) [11] and 21% of drug-resistant patients with almonertinib [12]. In terms of bypass activation, MET gene amplification is the most common resistance mechanism of osimertinib, accounting for 7%–24% of cases [13]. In patients on almonertinib, C797S and L718Q mutations formed the most common mechanism of EGFR-dependent drug resistance, while PIK3CA mutations, JAK2 mutations, BRAF mutations, and KRAS mutations, as well as HER2 amplification and FGFR3–TACC3 fusions, were the most common activation mechanisms of the bypass pathway [14]. However, second-generation sequencing (NGS) detection of the mechanism of drug resistance in tumors basically acts at the DNA level, so there remains a lack of studies comparing drug-resistant cells of third-generation EGFR-TKIs at the RNA-transcription level.

Thus, this study aims to compare the resistance mechanisms between osimertinib- and almonertinib-resistant cells, which is of great significance for improving the research of drug resistance and guiding treatment regimens.

## 2. Materials and Methods

### 2.1. Chemicals and Reagents

Almonertinib was donated by Hansoh Pharma (Jiangsu, China), and the drug powder was stored in a refrigerator at 4°C under sealed conditions and protected from light. Dimethyl sulfoxide (DMSO) was purchased from MP Biomedicals (Santa Ana, CA, USA).

### 2.2. Cell Lines, Cell Culture, and Almonertinib-Resistance Cell Induction

The human NSCLC with EGFR mutation cell lines H-1975 and HCC827 were obtained from the Shanghai Chinese Academy of Sciences cell bank (Shanghai, China). H-1975, established in July 1988, originated from a non-smoking female and harbors an L858R mutation in exon 21 along with a T790M mutation. HCC 827, established in March 1994, originated from a 39-year-old Caucasian female and features a deletion mutation in exon 19 [15, 16]. Both kinds of lung cancer cells were cultivated in RPMI-1640 medium (Thermo Fisher Scientific, Waltham, MA, USA) supplemented with 10% fetal bovine serum (Gibco Laboratories, Gaithersburg, MD, USA) and maintained in an incubator containing 5% CO_2_ at 37°C. Lung cancer cell lines were cultured with increasing concentrations of almonertinib beginning with 0.1 nM followed by a stepwise dose escalation every 2–3 generations up to 1 μM, and then the resistant cells (H-1975/AR and HCC827/AR) were maintained in 1 μM of corresponding compounds.

### 2.3. Cell Proliferation Assay (Cell Counting Kit [CCK]-8 Assay)

A cell counting kit (CCK)-8 kit (Dojindo Laboratories Co., Ltd., Kumamoto, Japan) was used to investigate cell proliferation. Cell lines (H-1975, HCC827) in the logarithmic growth phase were seeded into 96-well plates at a density of 5 × 10^3^ cells per 100 µL and cultured at 37°C and 5% CO_2_ in triplicate. After culturing for 24 h to enable the attachment of cells, cells were treated for a further 24 h with serial concentrations of almonertinib (i.e., 0.1, 1, 10, 100, 1, and 10 µM), vehicle control (dimethylsulfoxide), or blank control. 2 h before the termination of each time point, 10 µL of CCK-8 solution was added to each well and co-cultured with cells for another 2 h in a humidified environment containing 5% CO_2_ at 37°C. The optical density of each well was measured with a microplate reader (Bio-Rad Laboratories, Hercules, CA, USA) at the wavelength of 450 nm. The inhibition curves of almonertinib for each kind of cell were achieved.

### 2.4. Hematoxylin and Eosin (H&E) Staining

Cells were trypsinized and fixed in 4% paraformaldehyde (Beyotime Biotechnologies, Beijing, China) in phosphate-buffered saline solution (Thermo Fisher Scientific, Waltham, MA, USA). A histological examination was carried out using standard histological techniques. Fixed cells were stained with hematoxylin and eosin (H&E) stain (Beyotime Biotechnologies, Beijing, China), and observations were recorded under a light microscope.

### 2.5. Cell Cycle Assay

The cell cycle was investigated by flow cytometry analysis. Cells were harvested and fixed in 70% ethanol overnight, then combined with ribonuclease (50 μg/mL) and iodopovidone (50 μg/mL). After incubation for 30 min, samples were subjected to flow cytometry for analysis. Data were analyzed using FlowJo version 10.0 and then plotted.

### 2.6. RNA Transcriptome Sequencing of Almonertinib-Resistant Cell Lines

Total RNA was extracted using RNAex Pro reagent (Accurate Biotech, Hunan, China). Each group of cells sent three independent samples for detection, and Bioanalyzer 2100 was used to check the quality and quantity of RNA samples to ensure that the RIN of all samples was >7.0. Qualified RNA samples were sent to Novogene for library construction and sequencing. RNA libraries were prepared using the NEBNextUlltra TMRNA Library Prep Kit (Illumina, San Diego, CA, USA) and sequenced on the Illumina HiSeq platform.

### 2.7. Data Collection and Bioinformatics Analysis

A Gene Expression Omnibus dataset (GSE193258) was downloaded from the GEO database. The datasets contained the sequencing data of osimertinib-resistant cells H-1975/OR and HCC827/OR and corresponding control cells, respectively. The interactive GEO2R online tool (https://www.ncbi.nlm.nih.gov/geo/geo2r/) was used to identify the DEGs of osimertinib-resistant cells and their corresponding control cells according to a previously described method [17]. DEGs were defined based on an adjusted *p* value < 0.05 and |log2 (FC)| > 1.3.

### 2.8. Gene Ontology and Kyoto Encyclopedia of Genes and Genomes Analyses

To categorize the functional categories of DEGs, the KO-Based Annotation System (KOBAS 3.0) server was used to identify GO terms and KEGG pathways [18]. The Benjamini–Hochberg procedure and hypergeometric tests were used to define the enrichment of each term. GO information histograms and bubble maps of pathway information were drawn using an online bioinformatics tool (https://www.bioinformatics.com.cn) [19].

### 2.9. Survival Prognosis Analysis

The prognostic value of the mRNA expression of co-DEGs in lung adenocarcinoma patients was determined using the Encyclopedia of RNA Interactomes (ENCORI, http://starbase.sysu.edu.cn/index.php) database. Overall survival plots with hazard ratios (HRs) and log-rank *p* values were shown on the webpage. *p*-values of <0.05 were considered statistically significant.

### 2.10. Immune Cell Infiltration Assay

We used TIMER2 (Tumor Immune Estimation Resource, version 2, http://timer.comp-genomics.org) to assess associations between *IGFBP7* and immune cell infiltration by inputting “*IGFBP7”* in the “Gene_DE” query box. In this manner, we were able to obtain the specific immune cell infiltration associations in lung adenocarcinoma.

### 2.11. Quantitative Reverse Transcriptase PCR

Total RNA from cells was extracted using an EZ-press RNA Purification Kit (EZBioscience, Roseville, CA, USA), and cDNA was synthesized through reverse transcription using an Evo-MVRT Reagent Master Mix (Accurate Biotech, Hunan, China). The primers were designed according to the sequences in GenBank (http://www.ncbi.nlm.nih.gov/genbank/). The primer sequences were as follows: *IGFBP7* forward, ATCCCGACACCTGTCCTCAT, and reverse, CCCAGCCAGTTACTTCATGCT. The *IGFBP7* mRNA level relative to the *GAPDH* mRNA level was calculated using the comparative 2−*ΔΔ*Cq method.

### 2.12. Western Blotting Analysis

Cells were collected and then lysed in RIPA buffer for 30 min on ice. The supernatant was collected after centrifugation, and the protein concentration was determined through bicinchoninic acid (BCA) analysis (Beyotime Biotechnologies, Beijing, China). Equal amounts of protein were separated with SDS-PAGE (10%) before being transferred to PVDF membrane (Burlington, MA, USA). After blocking with 5% non-fat milk for 1 h at room temperature, the membranes were incubated overnight at 4°C with primary antibodies, namely *β-Actin* (Proteintech, Wuhan, China) and *IGFBP7* (Proteintech, Wuhan, China). Then, membranes were incubated with the appropriate secondary antibody for 1 h. Protein bands were detected using an enhanced chemiluminescence kit (Millipore, Burlington, MA, USA), and the protein expression was quantified using Image J software. *β-Actin* served as the internal control.

### 2.13. Gene Silencing by siRNA

The expressions of the *IGFBP7* genes were knocked down with small interfering RNA (siRNA) obtained from OBiO Technology (Shanghai, China). The siRNA was transfected into cells with Lipo8000 transfect reagent (Beyotime, Shanghai, China) and Opti-MEM (Thermo Fisher Scientific, Waltham, MA, USA) according to the manufacturer's protocol. The cells were used for experiments 48 h after transfection.

### 2.14. Cell Invasion Assay (Transwell Assay)

A transwell assay was performed to assess cell invasion capability. BioCoat cell culture inserts with a polyethylene terephthalate membrane (8-μm porosity) were placed in 24-well plates (BD Biosciences, Bedford, MA, USA). The upper chamber membranes were coated with Matrigel (Abwbio, Shanghai, China) and then incubated for 3 h at 37°C. Approximately 2 × 10^4^ H-1975/AR (si-NC) and H-1975/AR (si-*IGFBP7*) cells were added to the upper chamber in serum-free media, while 0.6 ml of complete culture medium was added to the lower chamber. After a 24-h incubation period, cells were washed twice with PBS, and any remaining cells were removed using cotton swabs. The cells on the bottom surface of the membrane were fixed with paraformaldehyde and stained with 0.1% crystal violet. The number of invaded cells on was recorded using an imaging device and counted under a light microscope.

### 2.15. Statistical Analysis

The data are expressed as mean ± standard deviation values and were analyzed with SPSS version 23.0 (IBM Corporation, Armonk, NY, USA). Differences between groups were analyzed using Student's *t* test. *p* < 0.05 was considered statistically significant.

## 3. Results

### 3.1. Construction and Validation of Almonertinib-Resistant Cells

**Figure 1:**
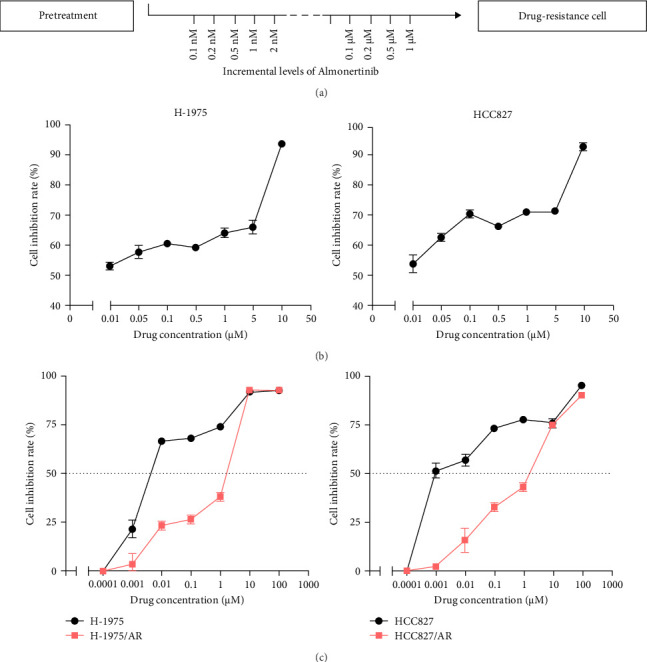
The culture method of almonertinib-resistant cells and the inhibition rate of different almonertinib on cells. (A) Incremental concentration culture method of almonertinib-resistant cells; (B) Drug sensitivity of H-1975 and HCC827 to almonertinib; (C) H-1975/AR and HCC827/AR to almonertinib drug sensitivity.

The drug sensitivity of primary cells to almonertinib was tested by CCK-8 cytotoxicity assay. The IC_50_ obtained for H-1975 cells was 1.9 nM, whereas that for HCC827 cells was 2.2 nM. The cell lines and EGFR mutation status required for the experiment are shown in Table 1. Almonertinib-resistant cells were cultured by increasing the drug concentration (Figure 1A). Results (Figure 1B, C) revealed that, with the increase in the concentration of almonertinib, the survival rates of both primary and drug-resistant cells were significantly reduced. The drug resistance coefficients were 833.58 for H-1975 and 631.95 for HCC827 (Table 1).

### 3.2. Morphological Changes in Almonertinib-Resistant Cell Lines

**Figure 2:**
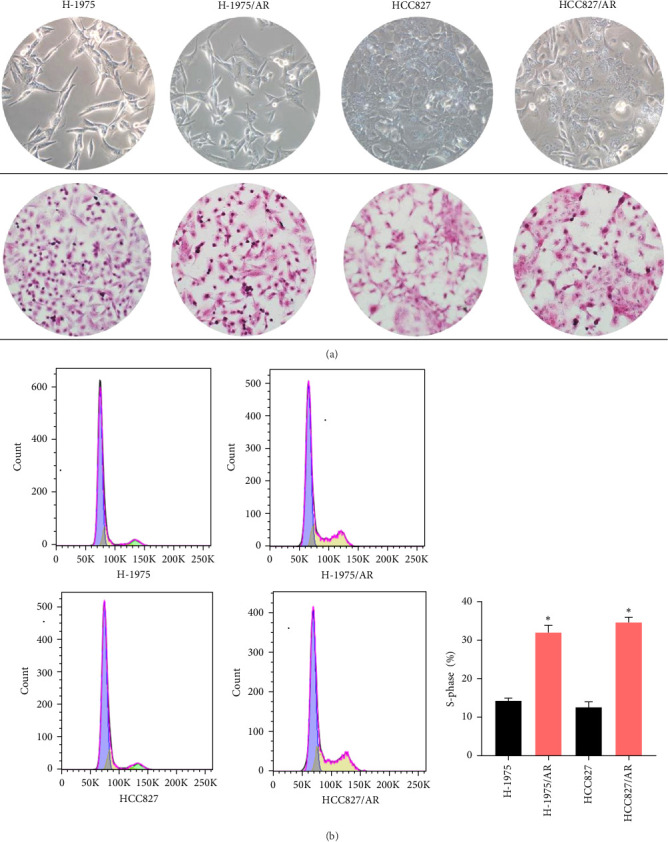
Morphological changes and cell cycle changes of cells before and after drug resistance. (A) Morphological changes and H-E staining changes of cell lines before and after drug resistance (×100); (B) Cell cycle changes of cell lines before and after drug resistance. “*⁣*^*∗*^” means *p* < 0.05, which means that the results are statistically significant.

The morphological changes and H&E staining changes of the two cell lines before and after drug resistance were observed under an inverted microscope (Figure 2A). The morphological changes of the H-1975 cell line were not obvious between before and after drug resistance, and H&E staining revealed that cell nuclei were enlarged compared to before. From the morphological observation of the HCC827 cell line, it was determined that the cell shape had changed from a rhomboid to a long spindle, and cell nuclei were larger than before.

### 3.3. Cell Cycle Changes in Almonertinib-Resistant Cell Lines

Compared to the primary cells, the number of S-phase cells in both groups of drug-resistant cells increased, and the difference was statistically significant (*p* < 0.05) (Figure 2B).

### 3.4. Transcriptome Sequencing Data and DEGs of Almonertinib-Resistant Cell Lines

A total of 12 samples of drug-resistant cells and primary cells were submitted for gene sequencing. The experimental results showed that a total of 220 DEGs were detected between H-1975 and H-1975/AR, among which 145 DEGs were downregulated and 75 were upregulated. Meanwhile, 736 DEGs in total were detected between HCC827 and HCC827/AR, among which 691 were downregulated genes and 45 were upregulated genes.

### 3.5. Screening of Overlapping DEGs of Osimertinib-Resistant Cells Identified from GEO Dataset

The dataset (GSE193258) for osimertinib-resistant cells (H-1975/OR, HCC827/OR) was obtained from GEO. A total of 2730 DEGs (1494 upregulated and 1236 downregulated) were identified in the H-1975/OR cell line, while 2307 DEGs (1702 upregulated and 605 downregulated) were identified in the HCC827/OR cell line.

### 3.6. GO Enrichment Analysis

**Figure 3:**
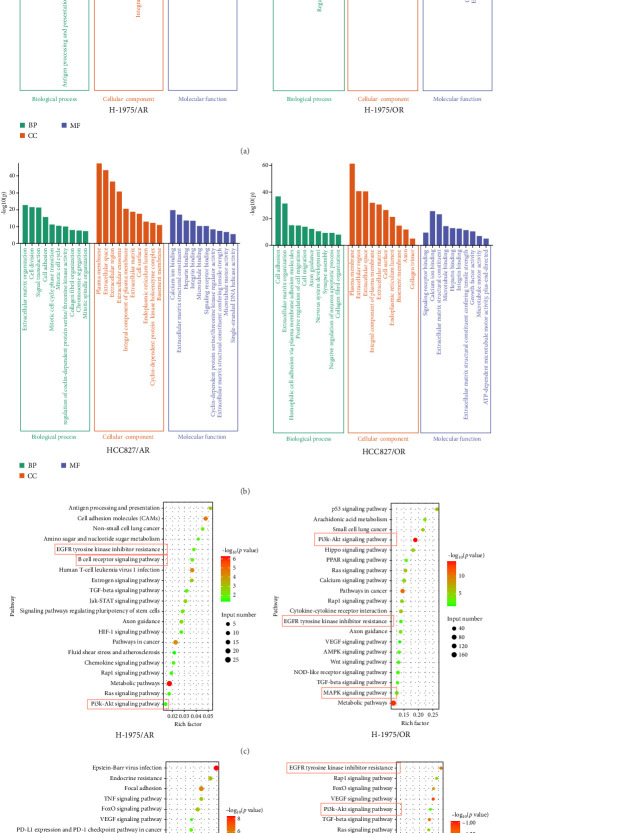
GO analysis and KEGG pathway analysis of the differentially expressed genes in drug-resistant cell lines. Barplot of GO analysis in H1975 (A) and HCC827 (B) resistance cell lines; Bubble plots of KEGG pathway analysis in H1975 (C) and HCC827 (D) resistance cell line. GO, Gene Ontology; KEGG, Kyoto Encyclopedia of Genes and Genomes.

The overlapping DEGs among the H-1975 resistant cell datasets were processed for GO enrichment analysis. As depicted in Figure 3A, the most-enriched biological processes for H-1975/AR were “negative regulation of cell population proliferation,” followed by “branching involved in blood vessel morphogenesis,” “negative regulation of protein binding,” “angiogenesis,” and “dendrite morphogenesis,” while those for H-1975/OR were “cell adhesion,” “mitotic cell cycle,” “external encapsulating structure organization,” “chromosome segregation,” and “signal transduction.” GO annotation for cellular components was also evaluated, and the primary cellular components of H-1975/AR were “integral component of membrane,” “endosome membrane,” “plasma membrane,” “ER to Golgi transport vesicle membrane,” and “extracellular region,” while the cellular components of H-1975/OR included “plasma membrane,” “extracellular exosome,” “integral component of plasma membrane,” “cell surface,” and “endoplasmic reticulum lumen.” For molecular functions for H-1975/AR, the DEGs were significantly enriched in the GO terms “protein binding,” “insulin-like growth factor binding,” “creatine kinase activity,” “peptidyltransferase activity,” and “transmembrane transporter activity,” while the DEGs were significantly enriched in “calcium ion binding,” “extracellular matrix structural constituent,” “integrin binding,” “signaling receptor binding,” and “cyclin-dependent protein serine/threonine kinase regulator activity” for H-1975/OR (Figure 3A).

The overlapping DEGs among HCC827-resistant cell datasets were also processed for GO enrichment analysis. For HCC827/AR, the DEGs were significantly enriched in the “antigen processing and presentation of exogenous peptide antigen via MHC class II,” “innate immune response,” “interleukin-27-mediated signaling pathway,” “positive regulation of immune response,” and “positive regulation of transcription, DNA-templated” biological processes. The DEGs were also significantly enriched in the “cytoplasm,” “nucleoplasm,” “membrane,” “trans-Golgi network membrane,” and “transport vesicle membrane” cellular components, as well as in the molecular functions of “metal ion binding,” “ATP binding,” “MHC class II receptor activity,” and “nuclear receptor binding.” (Figure 3B). For HCC827/OR, the DEGs were significantly enriched in the biological processes of “cell adhesion,” “homophilic cell adhesion via plasma membrane adhesion molecules,” “positive regulation of cell migration,” “cell migration,” and “synapse assembly,” as well as the cellular components of the “plasma membrane,” “extracellular space,” “extracellular matrix,” “cell surface,” and “endoplasmic reticulum lumen.” Finally, the DEGs were also enriched in the molecular functions of “calcium ion binding,” “extracellular matrix structural constituent,” “integrin binding,” and “growth factor activity” (Figure 3B).

### 3.7. KEGG Pathway Enrichment Analysis

Following the KEGG enrichment analysis of H-1975-resistant cells, the results of gene pathway enrichment showed that the resistance enrichment genes of H-1975/AR cells involved 83 meaningful pathway changes. These included the “Metabolic pathways,” “EGFR tyrosine kinase inhibitor resistance,” “Non-small cell lung cancer,” “PI3K-Akt signaling,” and “B cell receptor signaling” pathways (Figure 3C). For the H-1975/OR cells, 158 meaningful pathway changes were involved, including the “PI3K−Akt signaling,” “EGFR tyrosine kinase inhibitor resistance,” “cytokine−cytokine receptor interaction,” “MAPK signaling pathway,” and “calcium signaling” pathways (Figure 3C).

In the KEGG enrichment analysis of HCC827-resistant cells, HCC827/AR cell resistance enrichment genes were involved in 118 meaningful pathway changes, mainly comprising the “endocrine resistance pathway,” “EGFR tyrosine kinase inhibitor resistance pathway,” “B cell receptor signaling pathway,” “Wnt signaling pathway,” and “PD-L1 expression and PD-1 checkpoint pathway in cancer.” For HCC827/OR cells, the DEGs were involved in 158 meaningful pathway changes, primarily the “PI3K−Akt signaling pathway,” “MAPK signaling pathway,” “EGFR tyrosine kinase inhibitor resistance pathway,” “mTOR signaling pathway,” and “Wnt signaling pathway” (Figure 3D).

### 3.8. Differences in the Expression of EGFR Resistance Pathways

**Figure 4:**
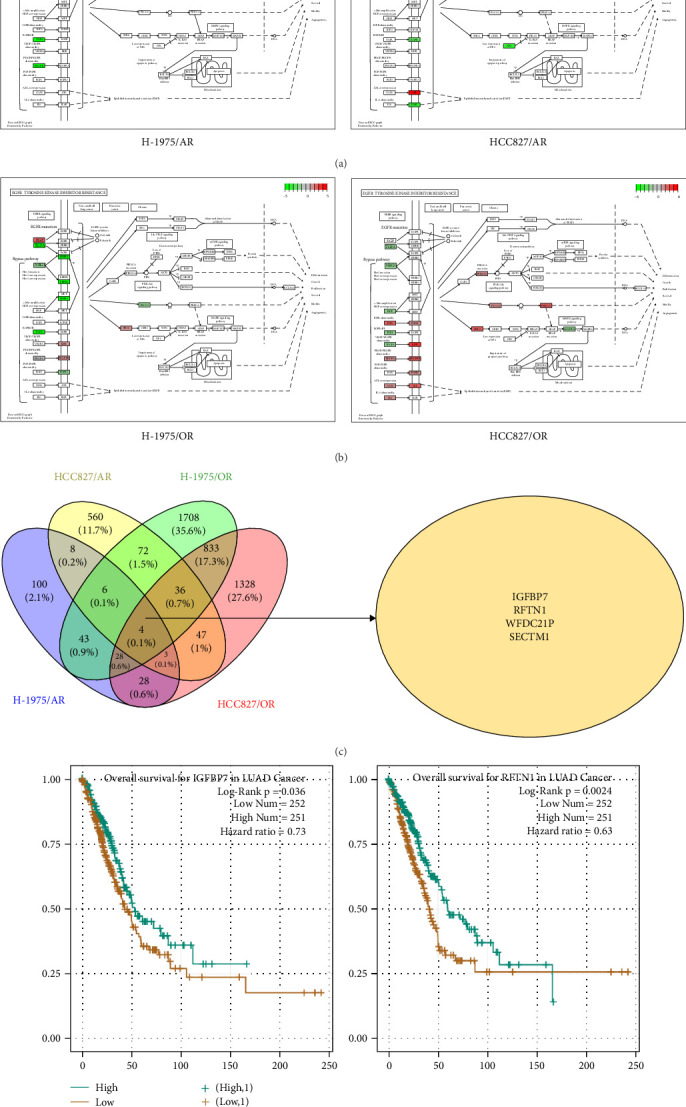
Analysis of drug resistance pathways and intersection of differential expressed gene sets in four drug-resistant cell lines and survival prognosis analysis of common differentially expressed gene (co-DEGs). Drug resistance pathway in almonertinib- (A) and osimertinib-resistant (B) cell lines. (C) Intersection of DEGs of four drug-resistant cell lines. (D) Survival prognostic analysis of co-DEGs (IGFBP7, RFTN1).

The difference in the EGFR-TKI resistance pathway among the resistance cell lines was further analyzed. Pathway analysis indicated that the EGFR-TKI resistance pathway was enriched in almonertinib- and osimertinib-resistance cell lines, respectively. H-1975/AR cells showed upregulation of AKT3 and HCC827/AR cells showed downregulation of EGFR expression and activation of the membrane protein gene *AXL* in the EGFR-TKI resistance pathway (Figure 4A).

Both osimertinib-resistance cell lines showed significant enrichment in the canonical EGFR-TKI resistance pathway. Separately, H-1975/OR cells showed upregulation of *SHC2* and *EGF*, while HCC827/OR showed upregulation of *SHC2* and *PIK3CD* (Figure 4B).

### 3.9. Intersection of DEGs of Osimertinib- and Almonertinib-Resistance Cell Lines

In order to determine the number of DEGs of osimertinib- and almonertinib-resistance cells, the Venny online tool (https://bioinfogp.cnb.csic.es/tools/venny) was used [19].

A total of 81 co-DEGs were found in common between H-1975/AR and H-1975/OR (Figure S1A), including *IGFBP7*, *MT1X*, *RFTN1*, *SDC2*, and *COL8A1*. Similarly, 90 co-DEGs were found in common between HCC827 and HCC827/OR (Figure S1B), including *IFI27*, *OAS2*, *MX2*, *IGFBP7*, and *ALDH1A1*. Moreover, taking the intersection of DEGs of four resistance cell lines, four genes (*IGFBP7*, *RFTN1*, *WFDC21P*, and *SECTM1*) were included. To clarify the impact of *IGFBP7*, *RFTN1*, *WFDC21P*, and *SECTM1* on the survival and prognosis of NSCLC patients, survival analyses were performed. As shown in Figure 4D, survival analyses indicated that high expression of *IGFBP7* and *RFTN1* (*green lin*e) is associated with a superior prognosis in NSCLC patients (*p* < 0.05), while the expression of *WFDC21P* and *SECTM1* is not associated with prognosis (*p* < 0.05) (Figure S2A, B).

### 3.10. Immune Cell Infiltration Analysis of *IGFBP7* and *RFTN1*

**Figure 5:**
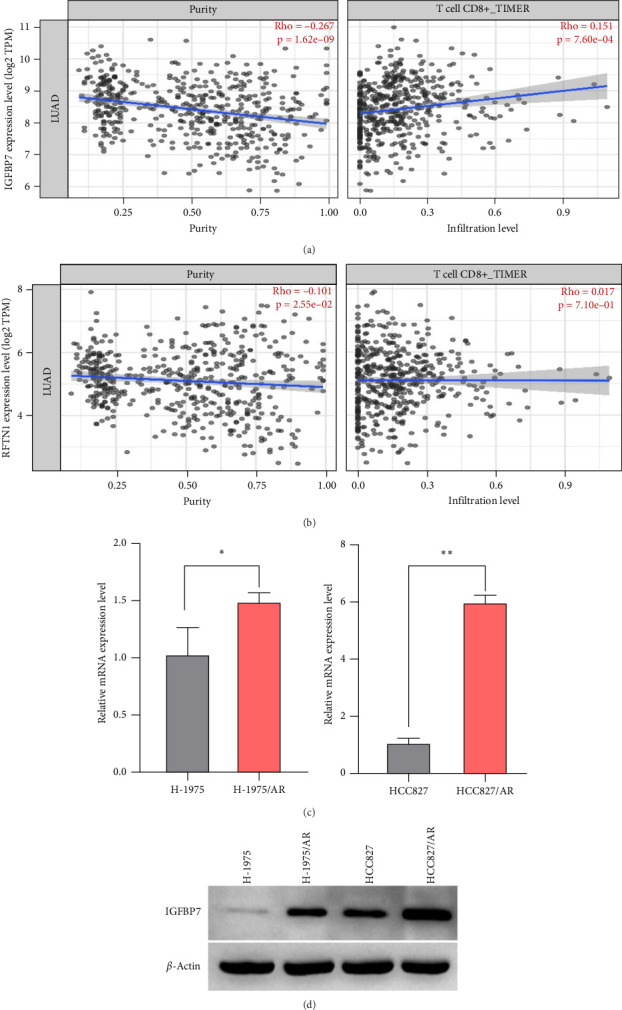
Analysis of the relationship between co-DEGs expression and infiltrating immune cells and validation of co-DEGs expression. The association between IGFBP7 (A) and RFTN1 (B) gene expression and CD8-positive (CD8+) T cells infiltration. (C) Validation of IGFBP7 gene high expression by qPCR in almonertinib-resistant cell lines. (D) Western blot verification of the high expression of IGFBP7 protein in almonertinib-resistant cell lines. DEGs, differently expresssed genes; qPCR, quantitative reverse transcriptase polymerase chain reaction. “*⁣*^*∗*^” means *p* < 0.05, which means that the results are statistically significant. “*⁣*^*∗∗*^” means *p* < 0.01, indicating that the difference is very significant.

The association between *IGFBP7* and immune cell infiltration was analyzed using the TIMER2 database. We found that the expression of the *IGFBP7* gene in lung adenocarcinoma had a significant positive correlation with the proportions of immune cells, including CD8+ T cells, Tregs, and macrophages (*p* < 0.05). For the *RFTN1* gene, there was no marked correlation between the proportion of immune cells and gene expression (*p* > 0.05) (Figure 5A, B; Figure S3A, B).

### 3.11. Verification of *IGFBP7* Expression Levels

The upregulation of gene expression was verified in parental and almonertinib-resistance cell lines through quantitative reverse transcriptase polymerase chain reaction (qPCR) and WB. We found that the almonertinib-resistance cell expressed a high level of *IGFBP7* in both RNA and protein levels (*p* < 0.05) (Figure 5C, D).

### 3.12. Verification of *IGFBP7* Knockdown in H-1975/AR Cells

**Figure 6:**
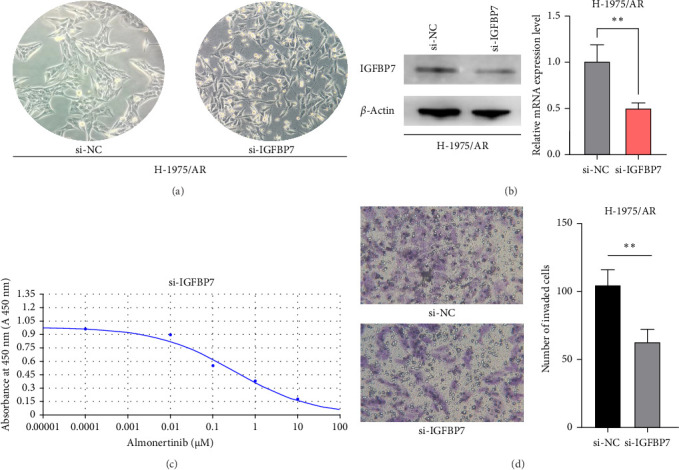
Verification of IGFBP7 gene function on drug-resistant cells. (A) Cell morphology of IGFBP7-knockdown H-1975/AR cells. (B) IGFBP7 expression levels in H-1975/AR cells after 48 hours of transfection. (C) IC50 curves of IGFBP7-knockdown H-1975/AR cells for almonertinib. (D) Invasive abilities of H-1975/AR cells after IGFBP7 knockdown. “*⁣*^*∗∗*^” means *p* < 0.01, indicating that the difference is very significant.

To examine the action mechanism by which resistant cells upregulate *IGFBP7* gene expression, we knocked down *IGFBP7* gene expression by transfecting siRNA into H-1975/AR cells for 48 h and examined its expression levels. Microscopic observation 48 h after transfection revealed that the morphology of H-1975/AR cells showed no alterations; however, there were increased numbers of falling floating cells, necrotic cells, and debris cells (Figure 6A). The qPCR and WB results displayed a downregulated expression level of *IGFBP7* genes in the knockdown group compared to the control group (Figure 6B).

### 3.13. Knockdown of *IGFBP7* Reduces H-1975/AR Cells Resistance to Almonertinib

The sensitivity of H-1975/AR cells to almonertinib was examined using a CCK-8 assay. The IC_50_ after 48 h of almonertinib treatment was determined (Figure 6C); the IC_50_ of almonertinib in H-1975/AR cells was 0.30 ± 0.02 µM after transfection, which was significantly lower than that of untransfected cells (Table 2).

### 3.14. Knockdown of *IGFBP7* Suppresses H-1975/AR Cell Invasion

To investigate the effect of *IGFBP7* on the invasion ability of three generations of EGFR-TKI-resistant cells, a cell invasion assay (transwell assay) was applied. As shown in Figure 6D, cell invasion was repressed after *IGFBP7* was downregulated for 48 h.

## 4. Discussion

Third-generation EGFR-TKIs, such as almonertinib and osimertinib, have been developed to overcome common EGFR-TKI resistance, but recent studies have shown that drug resistance remains a major challenge [20, 21]. Both H-1975 and HCC827 cell lines are primarily resistant to EGFR-TKIs, thus making them typical tumor models for studying drug resistance [22]. After the cells were treated with the medium containing almonertinib in increasing concentrations, the resistance factors were 833.58 and 631.95, respectively, indicating that these two cell lines have high resistance to almonertinib, with H-1975 cells showing a greater level of resistance than HCC827 cells. Drug resistance significantly altered the morphology and cell cycle of cells, with both H-1975/AR and HCC827/AR cell lines showing evident abnormal cell proliferation. These changes may be associated with significant alterations in intracellular protein expression and cell metabolism along with other microscopic cellular activities.

Combining high-throughput sequencing data from the almonertinib-resistant cell lines with osimertinib-resistant cell lines sequencing data from the GEO database, we further explored the potential molecular mechanisms after cell resistance. Notably, a greater number of downregulated genes were detected in drug-resistant cell lines, which may be associated with the reduced sensitivity of these cells to drugs. These findings suggest that gene-expression patterns significantly differ between drug-resistant cell lines and parental cell lines.

To explore the underlying resistance mechanisms, we conducted molecular pathway enrichment analyses of the cells. GO enrichment analysis indicated that the development of drug resistance in H-1975/AR and HCC827/AR cells may involve alterations in various cellular and biological processes—specifically, the resistance mechanism of H-1975/OR may involve changes in signaling pathways, while the resistance mechanism of HCC827/OR may involve changes in extracellular matrix interactions. Regarding KEGG enrichment analysis, we found that the classical drug-resistant EGFR tyrosine kinase inhibitor resistance pathway was enriched in all resistant cell lines. Furthermore, we found that the PI3K-Akt signaling pathway was enriched in HCC827/AR cells and that the B cell receptor signaling pathway and the MAPK signaling pathway were enriched in almonertinib- and osimertinib-resistance cell lines, respectively, when the screening threshold for mRNA was adjusted |log2 (FC)|>1. These findings suggest that the resistance mechanisms of different cell lines may be diverse and complex, involving alterations in multiple biological pathways and processes.

Consistent with previous studies, our analysis revealed a significant up-regulation of the classical PI3K/AKT resistance pathway in drug-resistant cell lines [23]. This pathway has been identified as a crucial component of the mechanism of resistance to EGFR-TKIs and plays a part in tumor progression and immune responses [24, 25]. Additionally, our findings indicate that EGFR expression was downregulated in HCC827/AR cells, while *AXL* expression was upregulated. *AXL* is known to be involved in tumor cell growth, metastasis, and drug resistance and has been shown to bind to members of the EGFR and HER families, including MET, to promote EGFR signaling and contribute to EGFR-TKI resistance [26]. These results suggest that the upregulation of *AXL* membrane protein levels may be an important factor contributing to EGFR-TKI resistance in HCC827/AR cells.

Both the *IGFBP7* and *RFTN1* genes were found to be differentially expressed in all four EGFR-TKI–resistant cell lines. Interestingly, further survival analysis indicated that elevated expression of *IGFBP7* was associated with a favorable prognosis in patients with NSCLC [27]. *IGFBP7* is a secreted protein with a molecular weight of 31 kDa that exhibits a high binding affinity for insulin. It functions as a tumor-suppressor by regulating various biological processes, such as cell proliferation, invasion, and angiogenesis [28]. Moreover, there is a link between *IGFBP7* and the tumor immune microenvironment. A previous study showed that increased *IGFBP7* expression is associated with poor prognosis and immune infiltration in gastric cancer [28]. In line with these findings, we performed immune infiltration analysis, which indicated that *IGFBP7* was strongly correlated with immune cells such as CD8+ T cells, Tregs, and macrophages in lung adenocarcinoma. Furthermore, the knockdown of the *IGFBP7* gene exhibited a tendency to restore cellular sensitivity to EGFR-TKIs, along with a decrease in invasive ability, implying that the *IGFBP7* gene plays a crucial role not only in maintaining cellular drug resistance but also in regulating cell proliferation and invasion abilities. These findings indicate that *IGFBP7* plays a particularly crucial role in regulating the growth and maintenance of drug resistance in third-generation EGFR-TKI-resistant cell lines. Although *IGFBP7* has been identified as a cancer suppressor, in this study it was associated with resistance to three generations of EGFR TKI drugs. Therefore, *IGFBP7* may play a different role in predicting lung cancer prognosis and drug efficacy. Anaplastic lymphoma kinase (*ALK*) is a similar target; NSCLC patients with *ALK* rearrangement have a poor prognosis, but also have a better response to ALK-TKIs, resulting in a better prognosis after treatment with them [29].

In addition to *IGFBP7*, the remaining differential genes (*SDC2*, *COL8A1*, *IFI27*, and *ALDH1A1*) were related to tumor drug resistance in the intersection of drug resistance differential genes. *SDC2* is a cell-surface proteoglycan that plays a crucial role in regulating various biological processes, such as cell growth, differentiation, adhesion, and migration [30, 31]. *COL8A1* is a gene that encodes for a component of the extracellular matrix, which has been implicated in tumor resistance and proliferation. Studies have reported overexpression of *COL8A1* in both lung and breast cancer, suggesting a potential role in tumorigenesis and disease progression [32, 33]. *IFI27* is known to be one of the most widely upregulated genes in cancer and plays a role in apoptosis, metabolism, the cell cycle, and tumor growth and suppression. Overexpression of *IFI27* has been shown to induce drug resistance in ovarian cancer cells [34, 35]. *ALDH1A1* has been identified as an oncogenic factor in many types of cancer and has been shown to amplify drug resistance in tumor cells through nucleotide metabolic pathways [36].

In summary, this study shed light on the distinct resistance mechanisms of almonertinib and osimertinib in NSCLC cells, which may have significant implications for targeted therapy in NSCLC. Nevertheless, it is important to note that this study was limited to the investigation of only two EGFR-TKIs, and further research into the resistance mechanisms of other EGFR-TKIs is warranted. Additionally, the current study was conducted exclusively in vitro, and clinical validation of our findings is still required.

## Figures and Tables

**Table 1:** Characteristics of cell lines and their changes in IC_50_ value.

Cell Line	Cancer types	EGFR mutation Status	*⁣* ^*∗*^IC_50_		Resistance index
			Pre-treatment (nM)	After treatment (μM)	
H-1975	Human lung cancer	L858R/T790M	1.90 ± 0.03	1.58 ± 0.36	833.58
HCC 827		19Del	2.20 ± 0.09	1.39 ± 0.04	631.95

*Note*: *⁣*^*∗*^IC50: half maximal inhibitory concentration.

Abbreviation: EGFR, epidermal growth factor receptor.

**Table 2:** Changes in IC_50_ values after knocking down the expression of *IGFBP7*.

Cell line	Group	*⁣* ^*∗*^IC_50_ (μM)
H-1975/AR	Control	1.58 ± 0.36
	si-IGFBP7	0.30 ± 0.02

*Note*: *⁣*^*∗*^IC50: half maximal inhibitory concentration.

## Data Availability

The data can be obtained upon request to the corresponding author.

## Supporting Information

Supporting Information Figure S1. Contained the intersection of gene sets between H-1975/AR and H-1975/OR and the gene sets between HCC827/AR and HCC827/OR cell lines. Figure S2. Contained the survival prognostic analysis of genes of WFDC21P and SECTM1. Figure S3. Contained the relationship between co-DEGs (IGFBP7, RFTN1) and infiltrated immune cells.
