# Supplementary material for: Comparison and Analysis of the Drug-Resistance Mechanism of Osimertinib- and Almonertinib-Resistant Cell Lines
Source: Anal Cell Pathol (Amst). 2025 Mar 10;2025:5578693. doi: 10.1155/ancp/5578693 (PMC11991788; doi:10.1155/ancp/5578693)
Supplement: Supporting Information — Figure S1. Contained the intersection of gene sets between H-1975/AR and H-1975/OR and the gene sets between HCC827/AR and HCC827/OR cell lines. Figure S2. Contained the survival prognostic analysis of genes of WFDC21P and SECTM1. Figure S3. Contained the relationship between co-DEGs (IGFBP7, RFTN1) and infiltrated immune cells. [file 5578693.f1.pdf]

Supplementary Figure S1

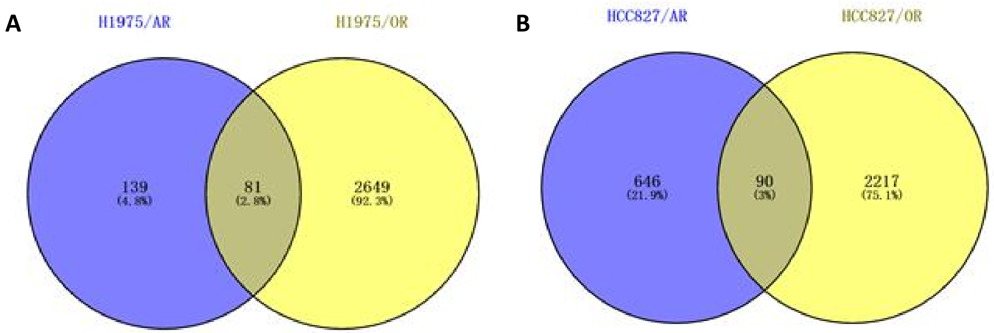

Supplementary Figure 1 Intersection of differential expressed gene sets between almonertinib- and osimertinib-resistant cell lines. (A) The intersection of gene sets between H-1975/AR and H-1975/OR. (B) The intersection of gene sets between HCC827/AR and HCC827/OR.

Supplementary Figure S2

A

B

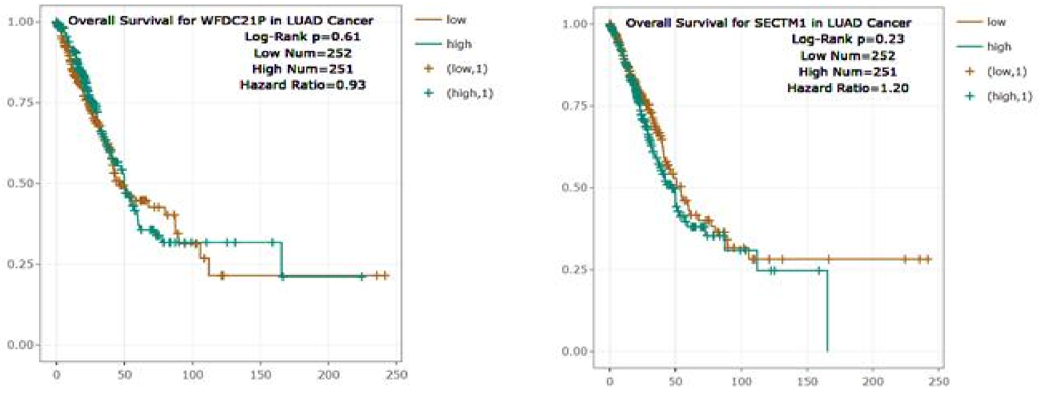

Supplementary Figure 2 Survival prognostic analysis of co-DEGs (*WFDC21P, SECTM1*). Survival prognostic analysis of *WFDC21P* (A) and *SECTM1* (B).

Supplementary Figure S3

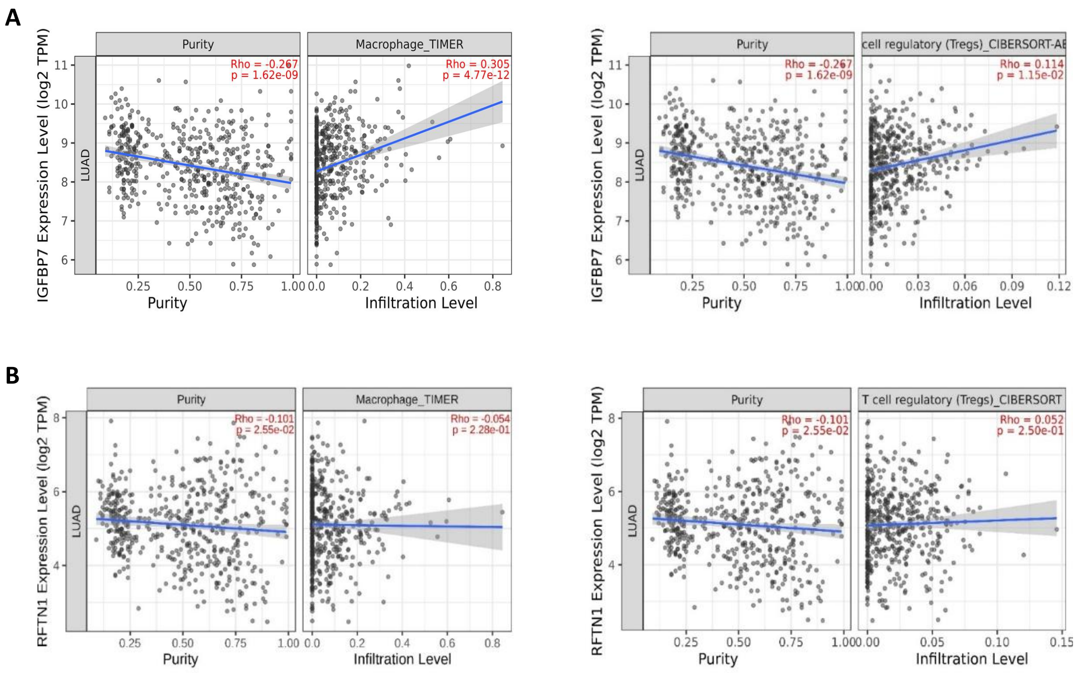

Supplementary Figure 3 The relationship between co-DEGs (*IGFBP7, RFTN1*) and infiltrated immune cells. Relationship between Treg cell (A) and Macrophage (B) and expression of *IGFBP7* ; Relationship between Treg cell (C) and Macrophage (D) and expression of *RFTN1.*
